# The stability and variability of serum and plasma fibroblast growth factor-23 levels in a haemodialysis cohort

**DOI:** 10.1186/s12882-018-1127-7

**Published:** 2018-11-14

**Authors:** Matthew J. Damasiewicz, Zhong X. Lu, Peter G. Kerr, Kevan R. Polkinghorne

**Affiliations:** 1 Department of Nephrology, Monash Medical Centre, Monash Health, Clayton, VIC Australia; 2 Department of Medicine, Monash University, Clayton, VIC Australia; 3 Monash Pathology, Monash Health, Clayton, VIC Australia; 4 Department of Epidemiology and Preventive Medicine, Monash University, Prahran, VIC Australia

**Keywords:** FGF-23, CKD-MBD, Haemodialysis, Phosphate

## Abstract

**Background:**

Serum fibroblast growth factor 23 (FGF-23) levels are markedly elevated in haemodialysis patients and have been linked to mortality outcomes. Small studies in health and chronic kidney disease, have demonstrated marked intra- and inter-individual variability in measured FGF-23 levels, and variable degradation in serum as compared to plasma samples. In end-stage kidney disease (ESKD), the intra- and inter-individual variability of FGF-23 levels, and the optimal collection methods remain poorly characterized. In this study we assessed the variability of FGF-23 levels in a cohort of stable haemodialysis patients. Secondly, in a subset of patients, we assessed the effects of different collection methods on measured FGF-23 levels.

**Methods:**

To assess the variability of FGF-23, pre-dialysis blood samples were collected over 3 consecutive weeks from 75 haemodialysis patients. The effects of different specimen collection methods were examined in a subset of patients (*n* = 23), with pre-dialysis blood collected into different tubes: plain (serum), EDTA (plasma) and EDTA with the addition of a protease inhibitor (EDTA-PI). All analyses were performed in the main cohort and repeated in each subgroup. Variability over a 3-week period was assessed using repeated measures ANOVA and random effects linear regression models. Intra-class correlation coefficients were calculated to assess agreement, and coefficients of variation were calculated to assess intra- and inter-individual variability.

**Results:**

Over the 3-week study period the mean FGF-23 levels were not significantly different in the serum (*p* = 0.26), EDTA (*p* = 0.62) and EDTA-PI (*p* = 0.55) groups. FGF-23 levels demonstrated marked intra- and inter-individual variability with a CV of 36 and 203.2%, respectively. In the subgroup analysis, the mean serum FGF-23 levels were significantly lower than the EDTA (*p* < 0.001) or EDTA-PI (*p* < 0.001) groups, however there was no difference in mean FGF-23 levels between EDTA and EDTA-PI (*p* = 0.54).

**Conclusions:**

The measured FGF-23 levels were significantly lower in serum as compared to plasma, and the addition of a protease inhibitor did not confer an additional benefit. Importantly in this cohort of ESKD patients, FGF-23 levels showed marked intra- and inter-individual variability. The routine measurement of FGF-23 in ESKD remains challenging, however this study suggests the plasma is the optimal collection method for FGF-23 analysis.

## Background

Fibroblast growth factor-23 (FGF-23) is a regulator of phosphate and mineral metabolism [1]. FGF-23 levels increase early in the course of chronic kidney disease (CKD), and precede the rise in serum phosphate and parathyroid hormone (PTH) level [2]. FGF-23 levels are inversely related to renal function and are markedly elevated in dialysis patients [3–5]. In large cohort studies, increased FGF-23 has been associated with increased risk of progression to end-stage kidney disease (ESKD) [6], and increased mortality in haemodialysis patients [7, 8]. These findings have generated a lot of interest in its role as a potential novel biomarker of CKD mineral and bone disorder (CKD-MBD).

Despite the emerging prominence of FGF-23 as a biomarker, the feasibility and clinical utility of routine FGF-23 measurements in CKD and ESKD cohorts remains unclear. This is in part due to a lack of standardization of the available assays and uncertainty about sample collection and processing. A number of different assays are currently available, measuring either cleaved (cFGF-23) or intact (iFGF-23) FGF-23 molecules [9]. In a small study in health and early CKD, FGF-23 levels were shown to degrade markedly in serum, and to a lesser extent in plasma [10]. A follow-up study also demonstrated significant inter- and intra-individual variability [11]. These studies highlight the ongoing uncertainty regarding optimal collection methods, processing and analysis of FGF-23 samples in health, CKD and ESKD.

In this prospective, observational study, we explored the issues around FGF-23 stability, collection and variability in a cohort of ESKD patients. First, we assessed the variability of serum FGF-23 levels over a 3-week period. Secondly, in a subset of patients, we assessed the effects of different collection methods on measured FGF-23 levels. We hypothesized that in a cohort of patients with ESKD, the variability of FGF-23 noted in general population cohorts is likely to be accentuated.

## Methods

### Study design

This was a prospective, observational study conducted over a 3-week period. Patients were recruited from satellite haemodialysis units at Monash Health.

To assess FGF-23 variability, pre-dialysis blood samples were collected from 75 haemodialysis patients over 3 consecutive weeks before the mid-week haemodialysis session. The effects of different specimen collection methods were examined in a subset of patients (*n* = 23) randomly chosen from the above cohort. Pre-dialysis bloods samples were collected over three consecutive weeks into different tubes: plain (serum), EDTA (plasma) and EDTA 4 ml tubes with 80 μl of added protease inhibitor (Sigma, MO, USA), (EDTA-PI), which was prepared and stored in accordance with the manufacturer’s specifications. Concurrent phosphate measurements were taken before each dialysis sessions, whilst other markers of CKD-MBD were collected at week-2. After collection, all blood samples for FGF-23 analysis were centrifuged and stored at − 70 °C within 3 h of collection until final analysis.

### Participants

Adult patients (age > 18 years) with ESKD, on maintenance haemodialysis were included in the study. Consent was obtained from all participants, and the study was approved by the Monash Health Human Research Ethics Committee. Patients were included in the study if they were stable on haemodialysis for three months or more. Patients with recent hospital admissions (< 1 month) due to infective complications were excluded. All patients were dialysed between 4 and 5 h, 3 times per week, using a Fresenius FX 80 high flux, polysulphone-helixone dialysis membrane. Clinical data and demographic details were obtained at the time of the blood collection. No changes were permitted in the dose or type of phosphate binder, nutritional vitamin D, calcitriol, calcimimetic, during the study period. The majority of patients received maintenance iron polymaltose on haemodialysis as per unit protocol. Given that there are no studies comparing different FGF-23 collection methods in ESKD patients, the determination of the study sample size was largely pragmatic and based on the maximum number of patients in whom we could process the samples in a timely manner. The cohort size is comparable to previous studies of repeated measures of FGF-23 in ESKD patients.

### Biochemical measurement

Common markers of CKD-MBD were analysed at Monash Pathology, all in serum except PTH, which was measured in EDTA plasma. Serum calcium and phosphate were measured by colorimetric methods using a Beckman DxC analyser, plasma PTH by immunoassay using a Beckman DxI and 25-hydroxyvitamin D [25(OH)D] using and Diasorin Liaison XL analyser. FGF-23 levels were measured in both serum and plasma in duplicates by an ELISA method using the 2nd Generation C-terminal FGF-23 ELISA assay (Immutopics, CA, USA). This assay measures both intact and large carboxyl- terminal fragments of FGF-23, and has a standard measuring range for calibration standards of up to 450 RU/mL (when read at 450 nm), extending up to 1400 RU/ml (when read at 620 nm), both were performed. The inter-assay CV was 1.4% at 302 RU/mL. Further manual dilutions were carried out as required in those with very high FGF-23 levels (> 1000 RU/ml) in order to obtain values in the optimal assay detection range.

### Statistical analysis

All analyses were performed in the main cohort (*n* = 75), with the variability analyses repeated in each of the subgroups (*n* = 23) for the separate collection methods (serum, EDTA plasma and EDTA-PI). Results were expressed as means with 95% confidence intervals unless otherwise stated. Agreement and reliability of the assays was assessed, individual intra-class correlation coefficient (ICC) was calculated using a one-way random effects model. Limits of agreement were also assessed using the Bland-Altman plots. Variability over the 3-week study period was assessed using repeated measures ANOVA and a random effects linear regression model. Coefficients of variation were calculated to assess the intra and inter-individual variability of FGF-23 measurements and were derived using the variance estimated from the regression models, while the mean was estimated from the study cohort.

*P* values of < 0.05 were considered statistically significant. All analyses were performed using Stata SE 13.0 (College Station, Tx,).

## Results

The detailed demographic and biochemical characteristics of the cohort are presented in Table 1.

**Table 1:** Main study cohort characteristics (*n* = 75) at week 2 unless otherwise stated

Study Population	
Age (mean, SE)	69.4 (1.2)
Gender (n, % male)	48 (66.7)
Cause of ESKD (n, %)	
DM	31 (41.3)
GN	23 (30.6)
Diabetes (n, %)	39 (52.0)
Dialysis Vintage (months, SE)	61.8 (6.3)
Dialysis duration (min, 95% SE)	269.2 (2.8)
Dialysis Access (n, %)	
AVF	67 (89.3)
AVG	5 (6.7)
Serum FGF-23 (mean RU/ml, SE)	
Week 1	1615.5 (396.4)
Week 2	1390.5 (318.0)
Week 3	1564.6 (376.7)
Phosphate (mean mmol/L, SE)	
Week 1	1.45 (0.05)
Week 2	1.46 (0.04)
Week 3	1.49 (0.05)
25(OH)D (mean nmol/L, SE)	70.7 (4.1)
PTH (mean pmol/L, SE)	32.6 (3.2)
Calcium (mean mmol/L, SE)	2.20 (0.02)
ALP (mean mmol/L, SE)	142.1 (10.1)
Cinacalcet (n, %)	26 (34.6)
Calcitriol (n, %)	43 (57.3)
Phosphate binders (n, %)	69 (92.0)

S*E* standard error, *ESKD* end-stage kidney disease, *DM* diabetes mellitus, *GN* glomerulonephritis, *AVF* arteriovenous fistula, *AVG* arteriovenous graft, *FGF-23* fibroblast growth factor 23, *PTH* parathyroid hormone, *25(OH)D* 25-hydroxyvitamin D, *ALP* alkaline phosphatase

### Variability

The mean serum FGF-23 levels at week 1–3 were; 1615.5 (95% CI, 825.6–2405.4), 1390.5 (95% CI, 756.8–2024.1) and 1564.6 (95% CI, 813.9–2315.2) RU/ml, respectively and there was no significant difference over the 3-week period (*p* = 0.26). The intra-class correlation coefficient (ICC) was 0.97 (95% CI 0. 96–0.98). The intra-individual CV was 36% (95% CI 32.1–40.3). There was marked dispersion of FGF-23 values across the cohort with an interquartile range (IQR) for the serum samples of (286–1310 RU/ml) and an inter-individual CV of 203.2% (95%, CI 172.8–238.8).

The mean phosphate levels at week 1–3 were; 1.45 (95% CI, 1.35–1.56), 1.46 (95% CI, 1.37–1.55) and 1.49 (95% CI, 1.40–1.59), with the levels not being significantly different (*p* = 0.48). The intra-individual CV for phosphate was 14.3% (95% CI, 12.7–16.0), while the inter-individual CV was 24.6% (95% CI 20.6–29.4). The inter-individual CV for a single measurement of PTH was 83.8%.

### Comparison of collection methods

**Fig. 1:**
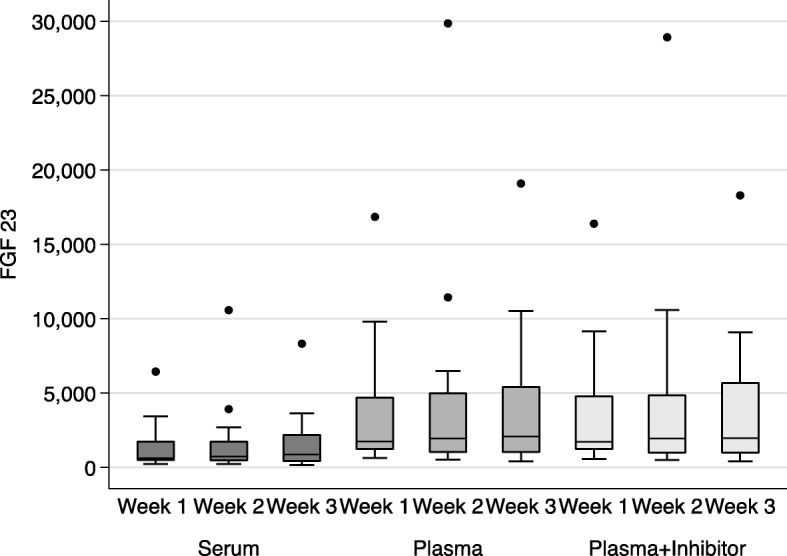
Box-plots demonstrating the serum, EDTA and EDTA-PI FGF-23 levels (RU/ml) over the 3-week study period (*n* = 23)

**Fig. 2:**
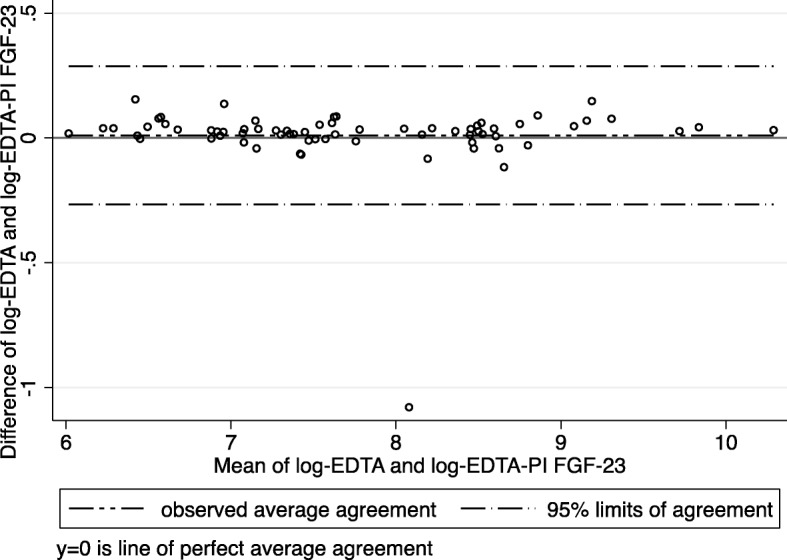
Bland-Altman plot demonstrating the agreement between EDTA and EDTA-PI FGF23 measurements (FGF-23 values are log-transformed)

The comparison of different collection methods was performed in a subgroup of 23 patients (Fig. 1). The mean FGF-23 levels in serum for weeks 1–3 were: 1228 (95% CI, 630–1826), 1500 (95% CI, 557–2443) and 1470 (95% CI, 704–2236) RU/ml, respectively. The mean FGF-23 levels in EDTA plasma for weeks 1–3 were: 3373 (95% CI, 1760–4987), 4146 (1478–6813) and 3874 (1995–5734) RU/ml, respectively. The mean FGF-23 levels in EDTA-PI plasma for weeks 1–3 were: 3317 (95% CI, 1751–4882), 4012 (95% CI, 1440–6584) and 3882 (95% CI, 2108–5657), respectively. FGF-23 levels were consistently lower in serum than in plasma from either of the EDTA (*p* < 0.001) or EDTA-PI (*p* < 0.001) groups (Fig. 1). The mean values between the EDTA plasma and EDTA-PI samples did not differ (*p* = 0.54), (Fig. 1). The differences between the results from the EDTA and EDTA-PI samples over a wide range of FGF-23 values (as depicted by the Bland-Altman plot, Fig. 2) were close to zero, indicating that the results obtained from these two samples were virtually identical.

### Variability – Subgroup analysis

#### Serum FGF-23 levels

The mean intra-individual CV was 34.2% (95% CI, 27.9–42.0). The mean values of FGF-23 for each individual were not different over the 3-week period, *p* = 0.19. The individual ICC for the serum FGF-23 levels over the 3-week period was 0.93 (95% CI 0.86–0.96, *p* < 0.001). The mean inter-individual CV was 121.9% (95% CI, 90.6–164%).

#### EDTA plasma FGF-23 levels

The mean intra-individual CV was 41.6% (95% CI, 33.9–51.1). The mean values of FGF-23 for each individual were not different over the 3-week period, *p* = 0.62. The ICC for the EDTA plasma FGF-23 levels over the 3-week period was 0.89 (95% CI, 0.80–0.95, *p* < 0.001). The mean inter-individual CV was 118.3% (95% CI 87.5–159.9%).

#### EDTA-PI plasma FGF-23 levels

The mean intra-individual CV was 42.6% (95% CI, 34.8–52.3). The values of FGF-23 for each individual were not different over the 3-week period, *p* = 0.55. The ICC for the EDTA plasma FGF-23 levels over the 3-week period was 0.88 (95% CI 0.78–0.94, *p* < 0.001). The mean inter-individual CV was of 114.7% (95% CI 84.7–155.2%).

## Discussion

Over a 3-week period, in a cohort of prevalent haemodialysis patients, we examined the agreement and reliability of FGF-23 measurements, as well as the intra- and inter-individual variability in FGF-23 levels. In a subset of patients, we examined the effects of different collection methods on measured FGF-23 levels and stability. Over a 3-week period there was no significant difference in the mean serum FGF-23 levels and serum FGF-23 levels demonstrated moderate intra-individual variability and marked inter-individual variability. Similarly, there was no difference in the mean levels of FGF-23 in any of the subgroups evaluating different collection methods (serum, EDTA plasma and EDTA-PI plasma). However, FGF-23 levels measured in serum were significantly lower in serum than in EDTA or EDTA-PI plasma. Finally, the addition of a protease inhibitor did not enhance the stability of FGF-23 levels (as compared to EDTA plasma alone) in this cohort.

FGF-23 levels increase early in the course of CKD and are markedly elevated by the time of ESKD [4, 8, 12]. The exact stimulus for this remains unclear, however it is likely that this is in part a physiological response to the rise in serum (and potentially local and bone) phosphate concentrations that occurs with a fall in the GFR. The increasing FGF-23 subsequently plays a pivotal role in the development of CKD-MBD through counter-regulatory effects on PTH and calcitriol synthesis [13]. Little is known about the degradation of FGF-23, however early studies suggest that it is not cleared by the kidneys, and the high levels seen in ESKD reflect intact FGF-23 [14, 15]. The breakdown products of FGF-23 remain poorly characterized, however these may also exert a yet unknown physiological effect [16, 17]. FGF-23 levels have been consistently associated with phosphate in ESKD, however in the absence of a direct phosphaturic effect, the function the supra-physiological FGF-23 levels seen in ESKD remains unclear [18, 19].

Little is known about the biological variability of FGF-23 in ESKD. Commercially available assays detect either iFGF23 or cFGF23 [9]. Small studies have shown reasonable agreement between the two assays in ESKD cohorts (in part likely due to the supra-physiological levels seen in these patients) [8, 15], however poor agreement at near physiological concentrations [11]. Furthermore a well conducted study in healthy individuals and early CKD showed that FGF-23 degrades more rapidly in serum than plasma samples, with higher values recorded by the cFGF-23 assay and substantial disagreement between the various assays [10]. In the same study the addition of a protease inhibitor conferred greater stability to the measured FGF-23 levels. Further studies in health and CKD have shown that both iFGF-23 and cFGF-23 show marked inter-individual variation [11]. It is also possible that the ratio of iFGF-23 and cFGF-23 in health and CKD is altered, although the pathophysiological implications of this are uncertain. There is no consensus as to the optimal collection methods or assay choice in measuring FGF-23, particularly in ESKD, where levels are markedly elevated and cannot be measured using currently available assays without serial dilutions. Currently most available assays measure cFGF-23, however it is possible that the different assays and collections methods may over or underestimate FGF-23 levels in different clinical contexts.

In our study we measured cFGF-23 levels and found no significant differences in the mean levels of over a 3-week period, and this result was replicated in the subgroup analysis. The ICC values showed a high agreement in measured values from week to week suggesting that the commercial assays perform satisfactorily in this cohort. However, the intra-individual CV remained high at 36%, compared to 14% for phosphate. In the subgroup analyses we found a pronounced and consistent difference between serum and plasma FGF-23 levels, with the measured serum levels being only approximately 30% of the measured plasma values. This may have significant implications for the measurement of FGF-23 levels, especially in cohorts where physiological concentrations are being measured. In contrast to other studies, we found that FGF-23 stability in plasma was not improved by the addition of a protease inhibitor, although it is possible that the supra-physiological levels of FGF-23 seen in this ESKD cohort obfuscated any potential benefit. Finally, our study demonstrated marked inter-individual variability of 203% for serum FGF-23 levels, (with similar findings in each of the 3 subgroup analyses), contrasted with 24% for phosphate and 84% for PTH. These findings confirm the large inter-individual variability of FGF-23 levels seen in normal and early CKD cohorts.

The use of FGF-23 levels both as a marker of CKD-MBD and treatment efficacy has been explored in clinical trials. In a post-hoc analysis of the EVOLVE trial, cinacalcet use was associated with a greater proportion of significant reductions in FGF23 levels [20]. A study comparing cinacalcet and low dose calcitirol to standard calcitriol therapy found higher FGF23 levels in the calcitriol treatment arm [21]. A study of commonly used phosphate binders found no apparent effect on measured plasma FGF23 levels [22]. FGF-23 has also been examined as a marker of dialysis efficacy in studies examining frequent dialysis protocols and high flux dialysers [23]. The high intra-individual variability in both plasma and serum observed in this study, suggests that FGF-23 levels should not currently be used to assess change or treatment efficacy in individual patients. Furthermore, the higher FGF-23 levels seen in plasma (as compared to serum), support evidence from earlier studies that FGF-23 degrades more rapidly in serum, and that plasma measurements are more reliable. These findings are particularly relevant for non-CKD and early CKD cohorts where near physiological FGF-23 levels are common, and where small variations in value may have greater clinical implications.

The marked inter-individual variability seen in this study makes the determination of a normal range and a potential threshold value for treatment initiation difficult. However, it does allow for hypothesis generating studies, which compare high (as opposed to low) FGF-23 levels and clinical outcomes. This is an issue that is shared by many markers of CKD-MBD most notably PTH. Despite the increasing number of epidemiological studies highlighting an association between FGF23, the clinical significance of a single FGF-23 level, as examined in many large cohort studies, should currently be interpreted with caution.

The strengths of our study included a well-defined cohort, with repeated measures conducted over a 3-week period, whilst controlling for all other clinical parameters. All specimens were collected and processed in manner to reflect “real world” conditions allowing them to be readily translated to clinical practice. The use of longitudinal regression models in to calculate the CVs, is statistically more robust, allowing for meaningful clinical interpretation and correlation of the study results. Our study has several limitations that need to be considered. Although we utilized the most commonly used cFGF-23 assay in clinical research, we did not specifically measure iFGF-23, which exerts the greater physiological effect. Given previous studies we would expect iFGF-23 levels to be lower given the degradation of FGF-23 following collection, although given supra-physiological levels seen in ESKD any clinical significance of this remains unclear. While both assays have been strongly associated with outcomes in CKD cohorts, more work is needed to define inter-assay variability and performance in real word clinical settings. This highlights the limitations of the current generation of assays in ESKD cohorts with high-expected FGF-23 levels, as the required dilutions may introduce measurement errors. We did not have data regarding phosphate intake and residual urine output both of which may confound FGF-23 measurements. Finally, although the study size compares favourably to other studies in the literature, greater patient numbers and longer study period may allow more informed conclusions to guide clinical practice.

## Conclusions

In conclusion, our study examined the variability and stability of FGF-23 in a cohort of stable haemodialysis patients. We found that EDTA plasma provides greater stability as compared to serum for FGF-23 analysis, and the addition of a protease inhibitor did not confer greater FGF-23 stability. The intra- and high inter-individual variability of FGF-23 is very high in patients with ESKD. The marked inter-individual variability seen is ESKD allows for future hypothesis generating studies, however this makes the determination of a clinically significant level difficult. Our results suggest that with careful collection and processing methods FGF-23 readings in plasma may be a useful biomarker of disease and treatment in selected patients. However, the relatively high intra-individual variability seen (in excess of 30%) makes it difficult to directly attribute any changes in measured FGF-23 levels to treatment, thus limiting its use as a reliable biomarker of treatment efficacy. These issues mirror the challenges seen with other markers of CKD-MBD such as PTH or phosphate measurements, but are unlikely to preclude the progressive integration of FGF-23 measurements into clinical practice. A greater understanding of FGF-23 physiology in ESKD, and new generation assays capable of detecting higher FGF-23 levels are needed before the widespread implementation of FGF-23 measurements in routine clinical practice.

## Abbreviations

ANOVA: Analysis of variance
CKD: Chronic kidney disease
CKD-MBD: Chronic kidney disease mineral and bone disorder
CV: Coefficient of variation
EDTA: Plasma collection tubes
EDTA-PI: Plasma collection tubes with the addition of a protease inhibitor
ELISA: Enzyme-linked immunosorbent assay
ESKD: End stage kidney disease
FGF-23: Fibroblast growth factor 23
GFR: Glomerular filtration rate
ICC: Intra-class correlation coefficient
IQR: Inter-quartile range
PTH: Parathyroid hormone
